# ZnO@ZIF-8 Core-Shell Structure Gas Sensors with Excellent Selectivity to H_2_

**DOI:** 10.3390/s21124069

**Published:** 2021-06-12

**Authors:** Ruonan Lv, Qinyi Zhang, Wei Wang, Yaojun Lin, Shunping Zhang

**Affiliations:** 1 School of Materials Science and Engineering, Wuhan University of Technology, Wuhan 430070, China; lrnrita0228@163.com (R.L.); ww18571471955@whut.edu.cn (W.W.); yjlin@whut.edu.cn (Y.L.); 2 School of Materials Science and Engineering, Huazhong University of Science and Technology, Wuhan 430074, China; pszhang@mail.hust.edu.cn

**Keywords:** ZIF-8, selectivity, H_2_, core-shell structure

## Abstract

As the energy crisis becomes worse, hydrogen as a clean energy source is more and more widely used in industrial production and people’s daily life. However, there are hidden dangers in hydrogen storage and transportation, because of its flammable and explosive features. Gas detection is the key to solving this problem. High quality sensors with more practical and commercial value must be able to accurately detect target gases in the environment. Emerging porous metal-organic framework (MOF) materials can effectively improve the selectivity of sensors as a result of high surface area and coordinated pore structure. The application of MOFs for surface modification to improve the selectivity and sensitivity of metal oxides sensors to hydrogen has been widely investigated. However, the influence of MOF modified film thickness on the selectivity of hydrogen sensors is seldom studied. Moreover, the mechanism of the selectivity improvement of the sensors with MOF modified film is still unclear. In this paper, we prepared nano-sized ZnO particles by a homogeneous precipitation method. ZnO nanoparticle (NP) gas sensors were prepared by screen printing technology. Then a dense ZIF-8 film was grown on the surface of the gas sensor by hydrothermal synthesis. The morphology, the composition of the elements and the characters of the product were analyzed by X-ray diffraction analysis (XRD), transmission electron microscope (TEM), scanning electron microscope (SEM), energy dispersive spectrometer (EDS), Brunauer-Emmett-Teller (BET) and differential scanning calorimetry (DSC). It is found that the ZIF-8 film grown for 4 h cannot form a dense core-shell structure. The thickness of ZIF-8 reaches 130 nm at 20 h. Through the detection and analysis of hydrogen (1000 ppm), ethanol (100 ppm) and acetone (50 ppm) from 150 °C to 290 °C, it is found that the response of the ZnO@ZIF-8 sensors to hydrogen has been significantly improved, while the response to ethanol and acetone was decreased. By comparing the change of the response coefficient, when the thickness of ZIF-8 is 130 nm, the gas sensor has a significantly improved selectivity to hydrogen at 230 °C. The continuous increase of the thickness tends to inhibit selectivity. The mechanism of selectivity improvement of the sensors with different thickness of the ZIF-8 films is discussed.

## 1. Introduction

As the global population grows, demand for energy significantly increases [1]. Hydrogen, as a clean energy, is considered an effective alternative to petroleum resources, attracting much attention in academia and industries [2,3,4]. Due to its flammable and explosive nature, hydrogen has safety risks in its production [5] and transportation [6], and therefore it is very necessary to monitor its leakage. According to the detection principle, hydrogen sensors are classified as metal oxide conductivity [7], solid electrolyte [8], contact combustion [9], quartz crystal microbalance (QCM) [10], and optical gas sensor [11]. Moreover, graphene oxide [12] and carbon nanotube [13,14] hydrogen sensors have been studied in recent years. Metal oxide semiconductors (MOSs) are widely used as a hydrogen detector [15,16] due to their low cost, ease of preparation, high stability, and high sensitive to the target gas. ZnO as a common metal oxide semiconductor has the advantages of being low cost, non-toxic, and having nanostructures easy to synthesize [17]. In recent years, it has become a very popular sensitive material for hydrogen detection [18]. Sensitivity, selectivity, stability, and speed of response are important criteria for evaluation of sensor performance. It is one of the most challenging research directions to improve the selectivity of sensors [19,20]. Common methods to improve the selectivity of MOS gas sensors include doping [21,22,23], microstructure control [24] and surface modification [25]. Qiao et al. prepared a sensor with high sensitivity, high selectivity and fast response to H_2_S with a concentration of 20 ppm by doping 0.2% Mo in BiVO_4_ [21]. Hiroyuki Abe et al. prepared a titanium oxide nanotube thin-film micro-scale gas sensor using a titanium microelectrode local anodization method, and modified the film with platinum nanoparticles to significantly improve its sensitivity to hydrogen and carbon monoxide gas. This sensor can detect hydrogen in the sub-ppm range [24]. Yang, et al. prepared a bi-layer structure sensor using a sol-gel method and magnetron sputtering technique, and the bi-layer structure consists of a pure tin oxide layer and a highly dispersed palladium nanoparticle, which significantly enhances the hydrogen sensing properties of SnO_2_ films [25]. Among all the methods to improve the hydrogen to other gas selectivity in gas sensors, surface modification is regarded as one of the most effective methods [26]. Xue et al. decorated the surface of an SnO_2_ sensor by introducing mesoporous SnO_2_, and in gas detection the modified sensor reacted to 1000 ppm hydrogen to 22.2 at 400 °C [27]. Meng et al. modified the surface of an SnO_2_ gas sensor with SiO_2_, and the modified sensor responds to 1000 ppm hydrogen up to 144 at 350 °C [28].

Metal-organic frameworks (MOFs) are highly active porous materials with high specific surface area newly developed in recent years [29]. The high specific surface area can absorb the gas in the pores, therefore improving the detection sensitivity. The tunable pore size and functional center can improve the detection selectivity through size exclusion or host-guest interaction [30]. The high porosity in MOFs enables the reversible absorption and release of guest molecules, thus ensuring the regeneration and recycling of detection [31]. Zeolite imidazole framework (ZIF) is one of the representative MOFs. Zn (II) or Co (II) is the metal center of ZIF, and the ligand is imidazole, which forms a three-dimensional framework through coordination [32,33]. ZIF-8 is considered to be one of the candidate materials to improve selectivity by covering metal oxide gas sensing layer [34]. ZIF-8 can effectively separate molecular mixtures and has a high permeability flux [35]. Meanwhile, it still has high stability at temperatures up to 500 °C [33]. Because of the strong adsorption capacity for gas, MOFs have been developed and applied in QCM sensors as sensor materials [10], and MOF sensors such as ZIF-8, ZIF-90 and HKUST-1 have a good stable response to acetone, CO_2_, hydrogen, etc., [36,37,38]. MOFs are also widely used in semiconductor gas sensors [20,39,40]. Zhou et al. successfully synthesized a core-shell structure of ZnO@ZIF-8 nanorod arrays using template method. In the tests on H_2_, NH_3_, ethanol, acetone and benzene, it was found that this ZnO@ZIF-8 sensor had a more significant response to hydrogen and ammonia than ethanol, acetone and benzene [39]. Ausama I Khudiar et al. used a similar method to coat ZIF-8 on the surface of ZnO nanorods to fabricate a gas sensor. In the test of H_2_ and C_6_H_6_, it was found that the selectivity of ZnO@ZIF-8 sensor to H_2_ has been significantly improved as compared with traditional sensors [20]. Wu et al. synthesized ZnO@ZIF-8 nanorods with core-shell structure by solution deposition method. The porous ZIF-8 shell improved the selectivity and sensitivity of the sensor to H_2_ [40]. However, the studies above only indicate that the application of MOFs to metal oxides for surface modification can greatly improve the selectivity and sensitivity of the sensor to hydrogen, while the mechanism of the selectivity improvement is still unclear. In addition, both gas sieving and catalysis were mentioned in the studies to improve selectivity of hydrogen gas sensors. It is well known that gas sieving and catalysis are closely related to the diffusion and interaction of gas molecules in MOFs [41], and therefore closely related to its diffusion distance. In other words, the thickness of MOF modified film has an important effect on the sieving and catalysis of gas molecules. However, so far few studies on the influence of modified film thickness on the selectivity of hydrogen sensors have been performed.

In this paper, ZnO nanoparticles were prepared by a homogeneous precipitation method, and ZIF-8 modified film was coated on the surface of the prepared ZnO by a hydrothermal method with the purpose of improving the selectivity of the ZnO gas sensors in hydrogen measurement. The ZnO@ZIF-8 core-shell composites with different thickness of ZIF-8 films were obtained by changing the reaction time of ZIF-8 modified film. By measuring hydrogen, ethanol and acetone at different operating temperatures, the effect of ZIF-8 thickness on the sensor hydrogen selectivity was evaluated, and the mechanism of selectivity improvement of the sensors with different thickness of the ZIF-8 films was also discussed.

## 2. Materials and Methods

### 2.1. Synthesis of ZnO NPs and ZnO NPs Gas Sensors

In this experiment, ZnO particles were prepared by a homogeneous precipitation method. 5.35 g of Zn(NO_3_)_2_·6H_2_O and 3.00 g of urea were put together with 100 mL of H_2_O. The beaker was sealed with polyethylene plastic film and magnetically stirred at 90 °C for 5 h to obtain qwhite precursor precipitation. The precipitate was cleaned in distilled water and ethanol for several times, followed by drying in an oven at 80 °C. The dried precursor was placed in a muffle furnace and heated to 500 °C at a heating rate of 2 °C/min and kept for 3 h. Then ZnO nano-particles (ZnO NPs) were obtained.

**Figure 1:**
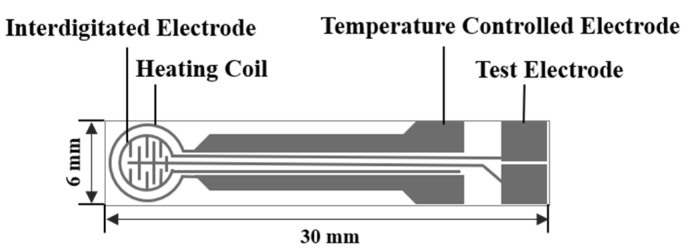
The schematic diagram of the TC-5010 sensor substrate.

The ZnO NPs prepared above and the printing oil (YY-1010, Wuhan Huachuang Ruike Tech. Co. LTD, Wuhan, China) were mixed at a mass ratio of 1:1, and ground in a mortar to form a white paste. The paste was applied to the sensor substrates (TC-5010, Wuhan Huachuang Ruike Co., Ltd., Wuhan, China) by screen printing technology. The schematic diagram of the substrate is shown in Figure 1. The staggered Pt electrode was printed on a substrate made of Al_2_O_3_ ceramic sheets. The newly printed sensors were flattened at room temperature for 10 min and dried at 60 °C for 1 h, then heated in a muffle furnace at 200 °C and 400 °C for 20 min, and finally heated to 600 °C for 2 h.

### 2.2. Synthesis of ZnO@ZIF-8 Gas Sensors

**Figure 2:**
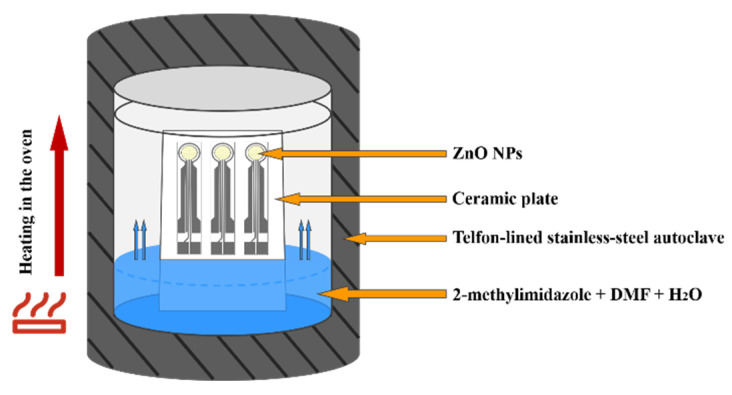
Schematic diagram of hydrothermal synthesis.

The 2-methylimidazole (0.04 g, 0.5 mmol) was placed in a Telfon-lined stainless-steel autoclave (50 mL) and dissolved with a N,N-Dimethylformamide (DMF)/H_2_O (16 mL, volume ratio 3:1) mixed solvent. The ZnO NPs gas sensors were fixed on a ceramic plate and placed vertically in the autoclave, as shown in Figure 2. The autoclave was placed in an oven for heating. As the temperature increased, the pressure inside the autoclave increased, and the liquid solvent gradually vaporized and the 2-methylimidazole reacted with ZnO NPs to form ZIF-8. The sensors were heated in 70 °C for 4 h, 8 h, 12 h, 16 h, 20 h and 24 h, respectively, then washed several times with ethanol. The sensors are pre-matured at 250 °C for 48 h before performance testing. The symbols of the sensors after different processing times are shown in Table 1.

### 2.3. Characterization

Phase analysis of the gas sensors was carried out by X-ray diffractometer (XRD, Empyrean, Almelo, The Netherlands). The morphology and microstructures of the products were observed by a field emission scanning electron microscope (SEM, JSM-7500F, Tokyo, Japan) with an accelerating voltage of 5 kV. The ZnO NPs and the ZnO@ZIF-8 were analyzed by transmission electron microscope (TEM, JEM-2100F, Tokyo, Japan). The element distributions of the products were analyzed by energy dispersive spectroscopy (EDS, JSM-7500F, Tokyo, Japan). With the integrated thermal analyzer STA449F3, the prepared ZnO@ZIF-8 was subjected to TG analysis in the temperature range of 25–900 °C at a heating rate of 5 °C/min. The full pore analysis of the products was carried out by the automatic specific surface area and porosity analyzer (BET, ASAP 2020M, Atlanta, GA, USA).

### 2.4. Measurement of Sensing Performance

The sensing performance test used the commercial SD-101 gas sensitivity test device (Wuhan Huachuang Ruike Technology Co., Ltd., Wuhan, China). All experiments were carried out at room temperature, 18–20 °C. In this experiment, the gas used in the gas distribution stage was dry synthetic air (the volume ratio of N_2_ and O_2_ is 4:1). The details of the test procedure can be found in our previous work [27]. The typical process was as follows: The dry air was introduced into the test chamber at a flow rate of 1000 mL/min, and the heating temperature was set as the test temperature. At this time, the gas sensor absorbed oxygen in the atmosphere to form oxygen ions (O_2_^−^, O^−^, O^2−^), and the resistances of the gas sensors gradually decreased to a stable state. Then, the flow of dry air and target gas were adjusted to reach the concentration required for the test, and the mixed gas was introduced into the test chamber. When the target gas contacted the sensor, it caused the sensors resistance to change and the sensors reached new resistance values after a period of time. After the sensors resistances were stable, dry air was introduced again at a flow rate of 1000 mL/min, and the sensors resistances gradually returned to their original state. The response (S) of the sensors is defined as Equation (1):

(1) $$S=\frac{R_{\mathrm{air}}}{R_{\mathrm{gas}}}$$

where *R*_air_ and *R*_gas_ are the resistances of the sensor in the air and test gas, respectively. The response coefficient is defined as Equation (2):

(2) $$D=\frac{S_{X}}{S_{0}}$$

where the *S_X_* is the response of the ZnO@ZIF-8 gas sensor and the *S*_0_ is the response of the untreated gas sensor in the same atmosphere. When *D* is greater than 1, the response of the gas sensor to the same target gas is increased, and when *D* is less than 1, the response of the gas sensor is decreased.

## 3. Results and Discussion

### 3.1. Characterization of the Gas Sensors

**Figure 3:**
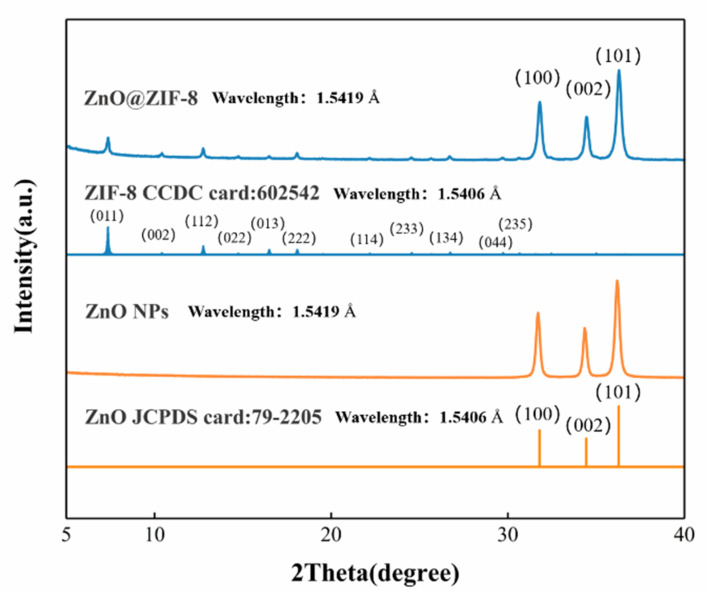
XRD patterns of the ZnO NPs and the simulated XRD patterns of ZnO@ZIF-8: The PDF standard card of ZnO and ZIF-8 are given under the respective measured patterns.

The XRD patterns of the ZnO@ZIF-8 and the ZnO NPs are shown in Figure 3. The XRD patterns of the ZnO NPs are in good agreement with the PDF standard card of ZnO (JDPDS card: 79-2205). According to the ZIF-8 PDF standard card [33], two crystalline phases of ZIF-8 and ZnO can be identified in the XRD of ZnO@ZIF-8 gas sensor.

**Figure 4:**
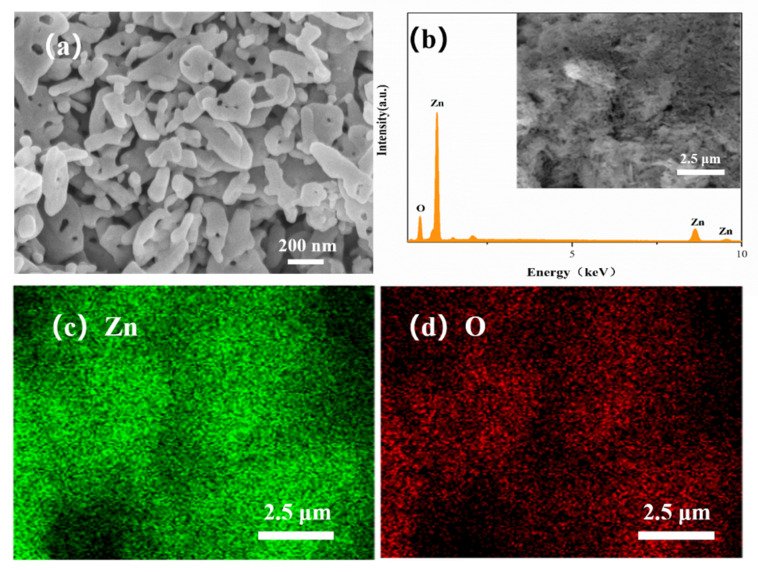
SEM scan (**a**) of ZnO NPs, EDS energy spectrum (**b**) and distribution of Zn (**c**), O (**d**) in surface scan image.

Figure 4a shows the surface morphology of the ZnO NP gas sensor. The ZnO NPs’ diameters are about 30–50 nm and some ZnO nanoparticles are bonded and formed into rods during high temperature sintering. The volatilization of printing oil leads to the uniform distribution of sintering holes with diameters of 10 nm to 200 nm in the sensors. Through EDS surface scan analysis (Figure 4b), the stoichiometric ratio of Zn and O is 47.27 at %: 52.73 at %, which is reasonably consistent with its chemical formula. The uniform distribution of Zn (Figure 4c) and O (Figure 4d) was verified by the EDS element analysis.

**Figure 5:**
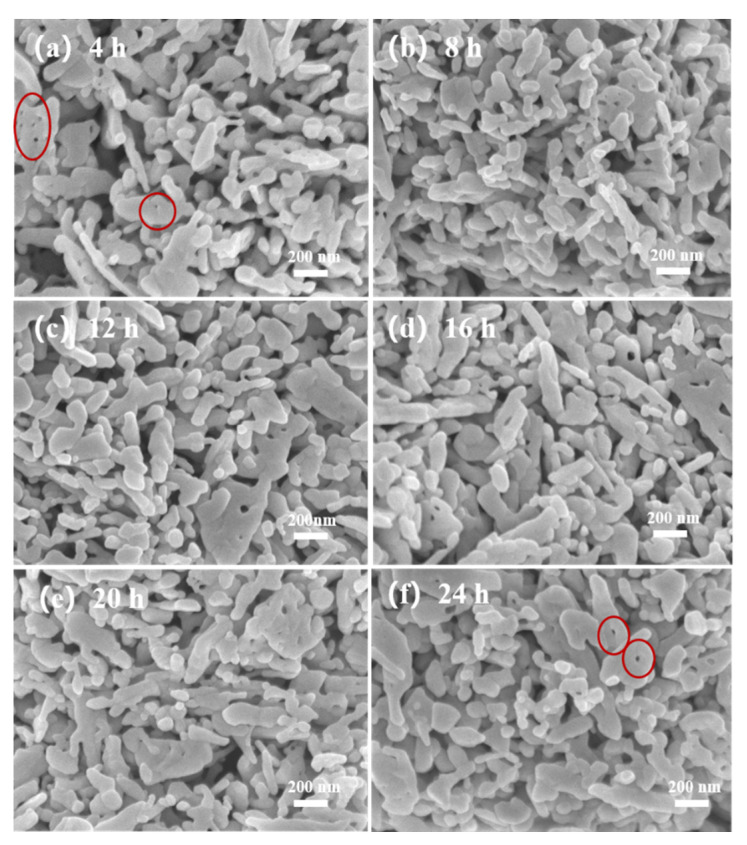
SEM of ZnO@ZIF-8 gas sensors after different growth times: (**a**) 4 h; (**b**) 8 h; (**c**) 12 h; (**d**) 16 h; (**e**) 20 h; and (**f**) 24 h.

**Figure 6:**
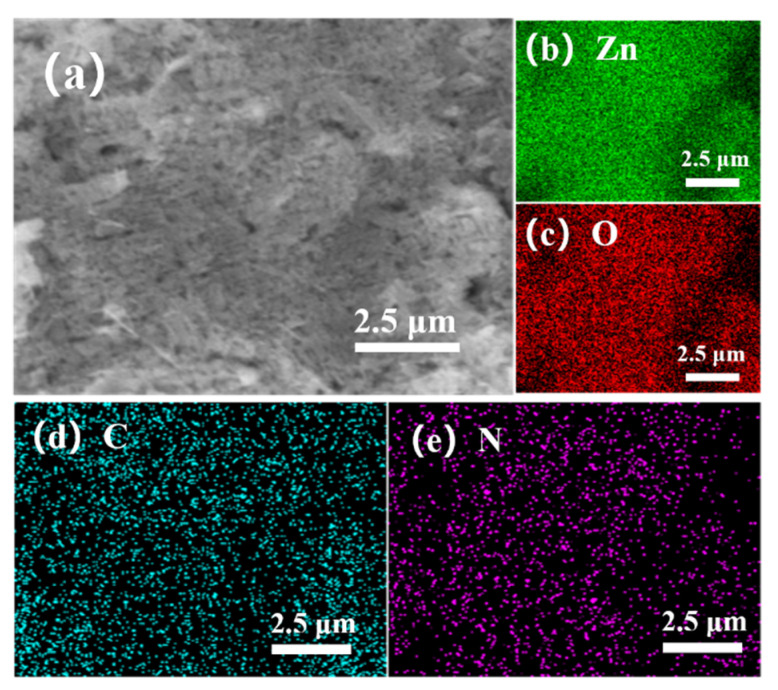
(**a**) SEM scan of 20-ZnO@ZIF-8, and distribution of (**b**) Zn, (**c**) O, (**d**) C and (**e**) N in surface scan.

Figure 5 shows the morphology of the ZnO@ZIF-8 sensors. It can be seen that the shape and size of ZnO NPs remain unchanged. In the sensor with a growth time of 4 h (Figure 5a), pinholes can be seen between the particles, which is due to the connection between the ZnO NPs caused by the growth of ZIF-8. In the sensor with 24 h growth time, the number of such pinholes decreased significantly, indicating that the gradual thickening of the ZIF-8 film allowed the pinholes to be filled. This can also be seen in the elemental analysis of the 20-ZnO@ZIF-8 NPs (Figure 6). It can be seen that C and N elements are evenly distributed in the surface, which indicates that new substances are formed on the surface of ZnO NPs. Combined with the XRD results (Figure 3), it can be concluded that ZIF-8 is formed on the surface. The core-shell microstructure of the ZnO@ZIF-8 sensors is the basis of the gas sensing test, and the dense ZIF-8 film is the key to verifying the influence of the pore size of ZIFs on the selectivity of the sensors [34]. Therefore, the sensors were analyzed by TEM.

**Figure 7:**
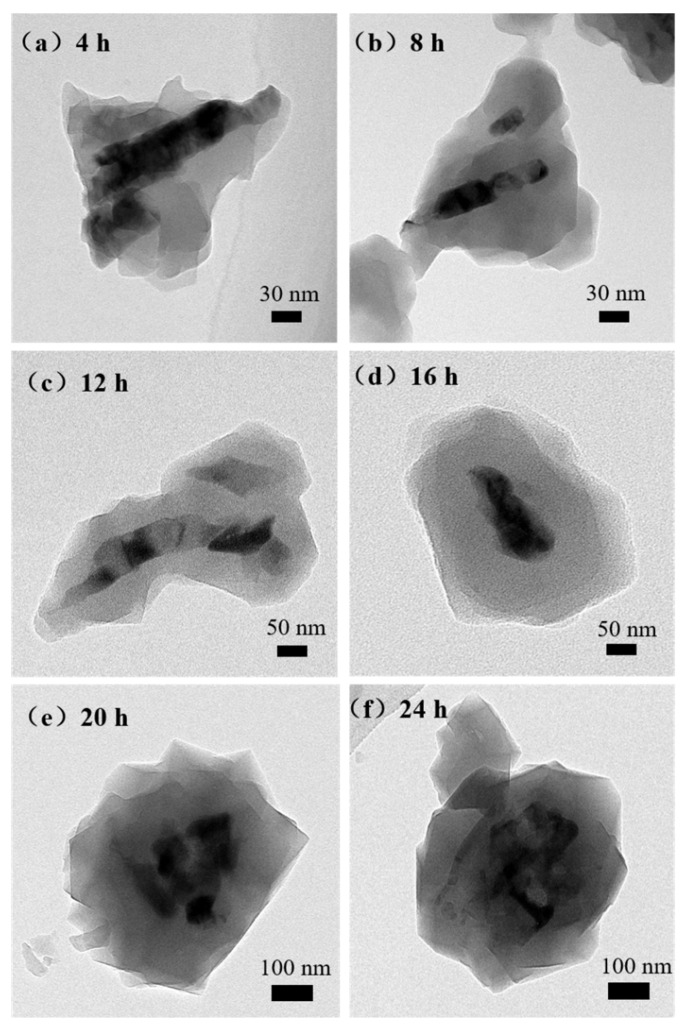
TEM images of the ZnO@ZIF-8 with different treating time: (**a**) 4 h; (**b**) 8 h; (**c**) 12 h; (**d**) 16 h; (**e**) 20 h; (**f**) 24 h.

The products were analyzed by TEM to further confirm whether the synthesized ZnO@ZIF-8 materials had core-shell microstructure. An irregular film can be clearly seen on the ZnO NPs surface by transmission electron microscope, as shown in Figure 7. The thickness of ZIF-8 in the 4-ZnO@ZIF-8 was about 50 nm, while that of the 8-ZnO@ZIF-8 was about 60 nm, but some of the ZnO NPs were not completely wrapped. This result indicated that although the thickness of the ZIF-8 film increased with time, the film could not form an integral and dense core-shell structure in a relatively short period of time. When the time reaches 12 h, the ZIF-8 film had wrapped all the ZnO NPs and the thickness of the film gradually became even. After 16 hours’ treatment, the ZIF-8 film reached 100 nm, the average thickness of the ZIF-8 in the 20-ZnO@ZIF-8 was 130 nm, and the thickness of the ZIF-8 film in 24-ZnO@ZIF-8 reached 150 nm. It can be seen that with the increase of treating time, the size of the ZnO particles gradually decreased, and the edges of the ZnO particles began to blur, showing irregular boundaries. The Zn^2+^ source for ZIF-8 was provided by ZnO NPs, and 2-methylimidazole was the ligand of the ZIF-8 [39].

**Figure 8:**
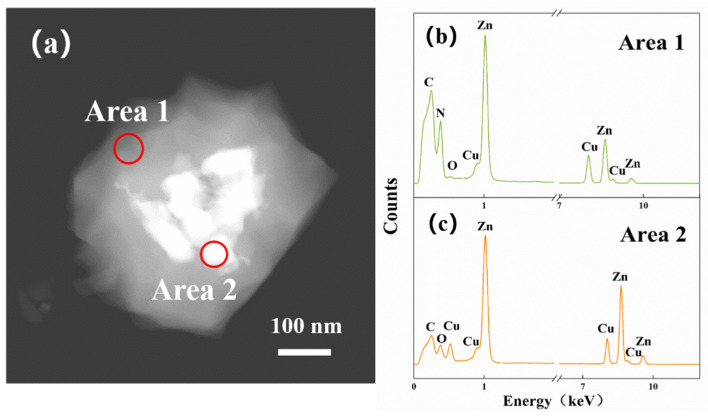
TEM image of 20-ZnO@ZIF-8 (**a**) and elemental energy spectrum of the shell (**b**) and the core (**c**).

Figure 8a is a TEM dark field image of the 20-ZnO@ZIF-8 gas sensor. The bright area represents the wrapped ZnO particles in the material. Two points with quite different brightness are selected for EDS analysis. Figure 8b,c show the EDS spectrum corresponding to areas 1 (shell) and area 2 (core). N and C are abundant in the composition in area 1, but the composition in area 2 dominantly consists of ZnO. Based on the XRD, SEM, TEM and EDS results, it can be confirmed that the synthesized product is ZnO@ZIF NPs, and with the extension of the growth time of the ZIF-8, a dense core-shell structure is gradually formed.

**Figure 9:**
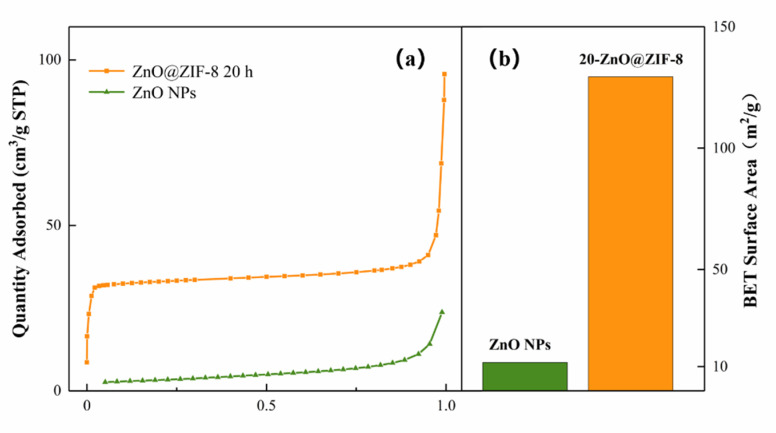
Adsorption/desorption isotherms of ZnO NPs (**a**) and 20-ZnO@ZIF-8 and BET specific surface area (**b**).

The gas adsorption capacity and specific surface area of the ZnO@ZIF-8 NPs composite were verified by nitrogen absorption/desorption isotherm and BET specific surface area tests. From the results (Figure 9), it can be seen that the ZnO@ZIF-8 have superior adsorption capacity and specific surface area than the ZnO NPs. The specific surface area of the 20-ZnO@ZIF-8 material is 129.4 m^2^/g, while the specific surface area of the ZnO NPs is only 11.7 m^2^/g, indicating that the specific surface area of the ZnO@ZIF-8 significantly increases by the growth of the ZIF-8 film on the surface of the ZnO nanoparticles. Therefore, the ZnO@ZIF-8 composite can be used as a high-efficiency absorber for gas trapping and pre-enrichment, which provides a good foundation for improving the sensitivity of MOS gas sensors [42].

**Figure 10:**
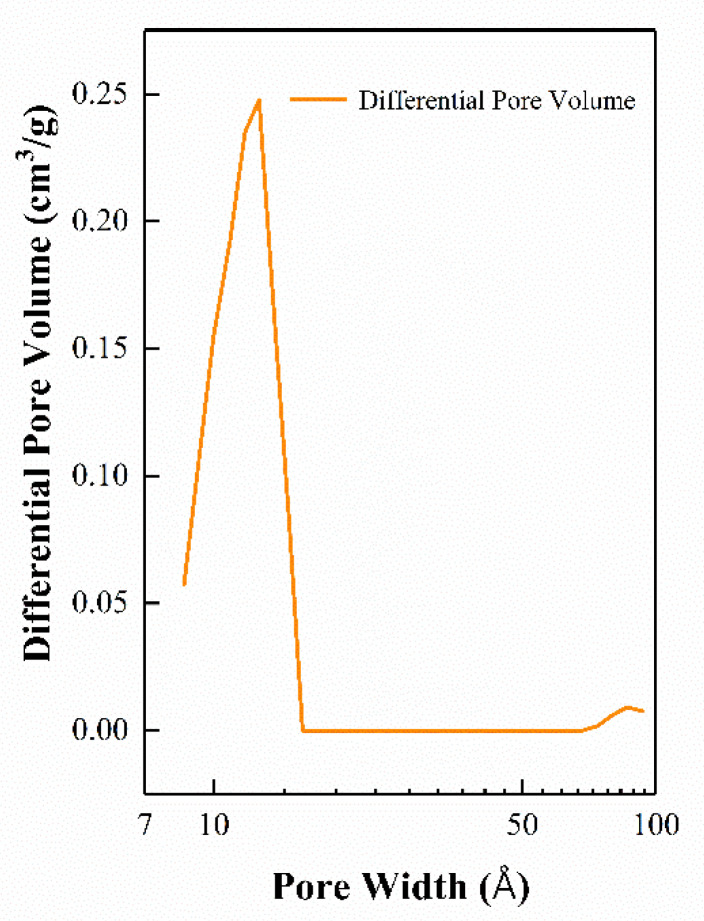
Pore size distribution of the 20-ZnO@ZIF-8 gas sensor.

Taking the 20-ZnO@ZIF-8 sensor as an example, the pore size distribution is shown in Figure 10. The pore size distribution diagram shows that the micropore structure of the ZnO@ZIF-8 prepared in this experiment exhibits a narrow distribution of pore size in the range of 11–12 Å. MOFs can sieve gas molecules according to the pore size, and narrow pore size distribution can better restrict gas molecules with large dynamic media to permeate the MOF’s film. In contrast, gas molecules with a dynamic diameter of less than 11–12 Å can enter the gas sensor relatively easily to react with the sensitive materials.

**Figure 11:**
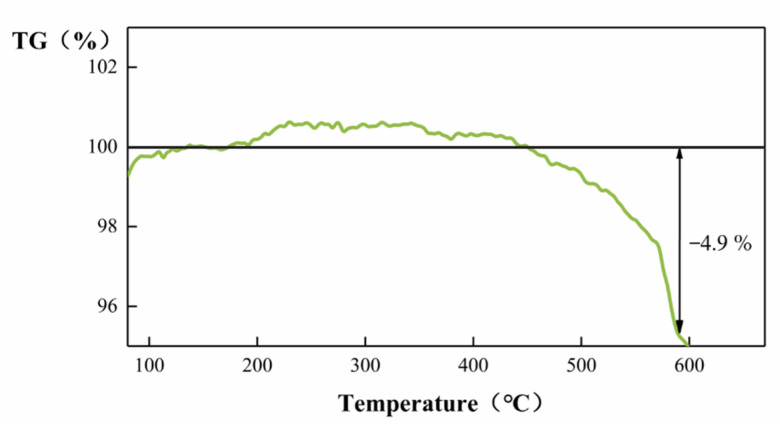
TG curve of the ZnO@ZIF-8 composite.

The thermal stability of the 20-ZnO@ZIF-8 composite was tested by a comprehensive thermal analyzer. The Figure 11 shows that the gas sensor can maintain good thermal stability under 400 °C. When the temperature rose to 450 °C, the sensor began to lose weight. The weight loss reached 4.9% at 590 °C. The sensor has good thermal stability below 400 °C. Considering the actual test conditions of the sensor, the maximum test temperature in this experiment is 290 °C.

**Figure 12:**
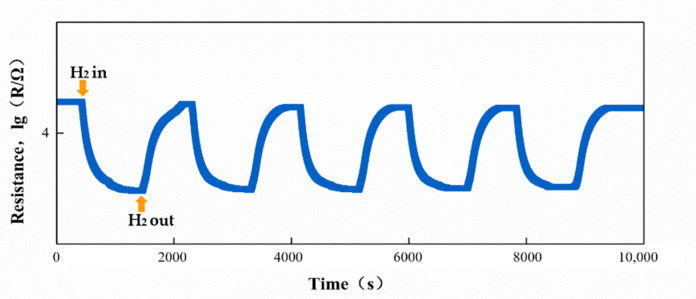
Cycle curve of the 12-ZnO@ZIF-8 gas sensor in hydrogen test.

Stability is one of the important parameters to evaluate the performance of gas sensors. The traditional ZnO gas sensor can be tested multiple times due to its stable structure and physical and chemical properties [17]. However, the physical and chemical properties of ZIF-8 are not as stable as ZnO due to the diversity of the structure. In order to verify the stability and the reliability of the test data, taking the 12-ZnO@ZIF-8 gas sensor as an example, a cyclic test was performed on H_2_ (1000 ppm) at 250 °C, and the transient response curve is shown in Figure 12. It can be seen from the transient response curve that the ZnO@ZIF-8 gas sensor can perform gas adsorption and desorption in multiple cycles. In terms of the response, the response of the gas sensor is stable at about 2.0, and the response time and recovery time are relatively short. The resistance of the sensor during the second test was slightly lower than in the first test, and the baseline resistance gradually stabilized after the third cycle. This is due to the continuous adsorption and desorption of the sensor leading to incomplete desorption in the working process. The phenomenon has also been reported by other researchers [43]. This shows that the sensor has good stability.

### 3.2. The Resistance of the Sensors in Air

**Figure 13:**
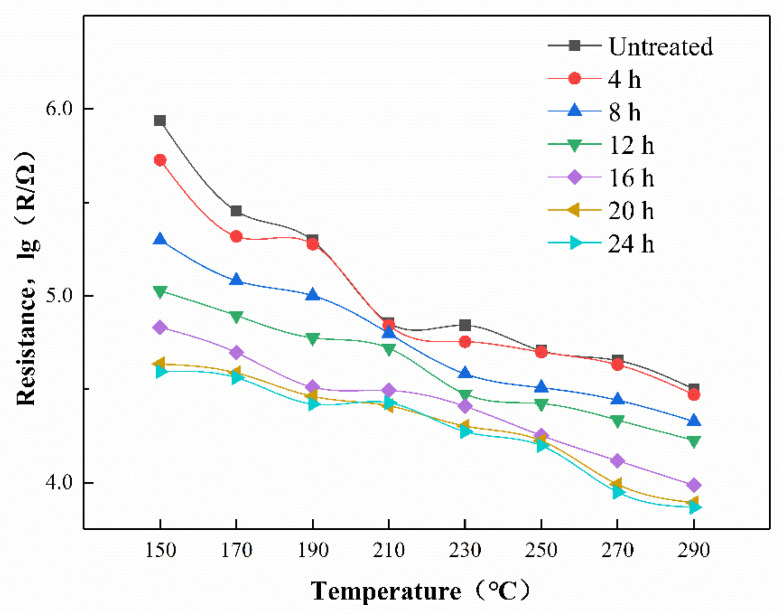
The temperature-resistance curve of gas sensors.

The temperature-resistance curve of gas sensor in the air is shown in Figure 13. As the operating temperature increases, thermal excitation causes the increase of carriers in the semiconductor sensor, and the resistance of the gas sensor shows a downward trend [28]. ZnO is a kind of semiconductor material. Under thermal excitation conditions, the carrier of the sensor increases, leading to an increase in conductivity and a decrease in resistance. Under constant temperature conditions, the resistance of a gas sensor mainly depends on the amount of oxygen absorbed [44]. O_2_ attached to the surface of the gas-sensitive material absorbs electrons in the conduction band of ZnO, resulting in a reduction in the number of carriers and an electron depletion layer. The electron depletion layer of the gas sensor becomes thicker, and the resistance of the gas sensor becomes higher. Due to the dense structure of ZIF-8 hindering the contact between oxygen and the ZnO NPs, the air resistance of the gas sensor compared to the ZnO NPs decreased. As the growth time of the ZIF-8 film increased, the ZIF-8 film on the surface of the ZnO gas sensor completely wrapped the zinc oxide particles and gradually thickened. The thicker ZIF-8 film reduced the chance for O_2_ to pass smoothly and contact the ZnO particles, and the amount of oxygen adsorbed on the surface of the ZnO particles decreased. Therefore, as the growth time of the ZIF-8 film increased, the resistance of the gas sensor in air gradually decreased.

### 3.3. Gas Sensing Measurements

**Figure 14:**
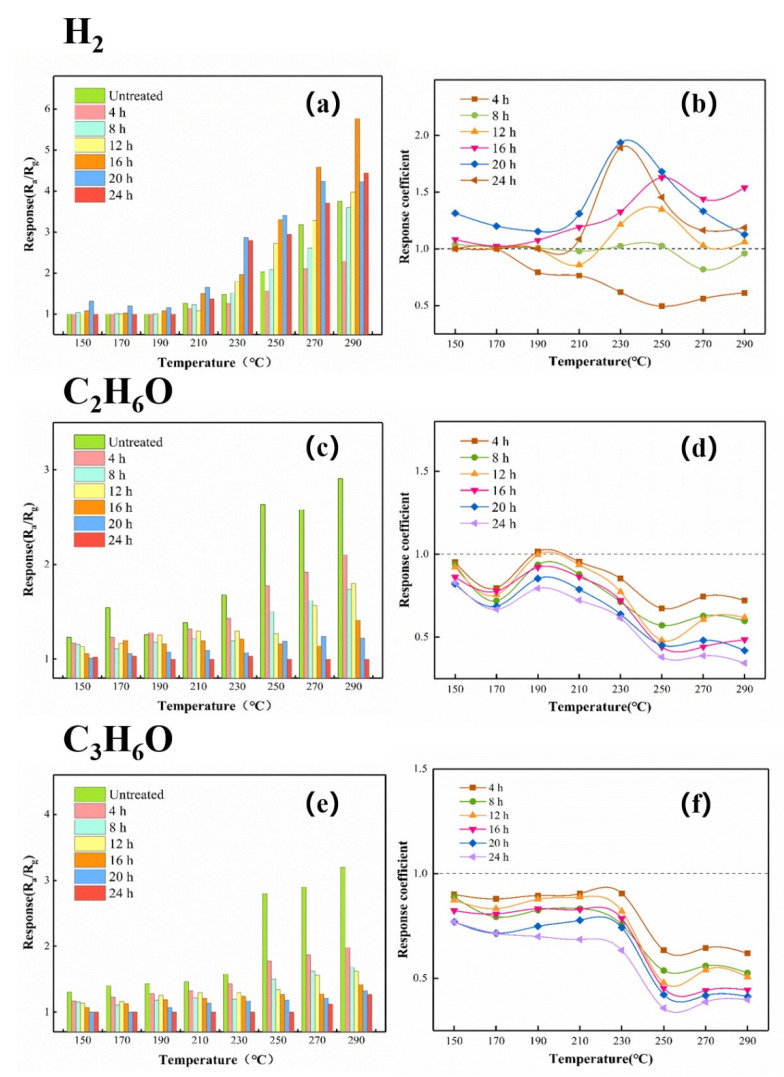
The responses and response coefficients of the gas sensors to different gases: (**a**,**b**) 1000 ppm H_2_, (**c**,**d**) 100 pm C_2_H_6_O, and (**e**,**f**) 50 ppm C_3_H_6_O.

In order to correlate the thickness of the ZIF-8 film to the selectivity of the ZnO@ZIF-8 gas sensor, a series of experiments were designed to test gases of different molecular sizes (1000 ppm H_2_, 100 ppm C_2_H_6_O, 50 ppm C_3_H_6_O). The responses of the gas sensor to each gas are shown in Figure 14. Below 200 °C, the gas sensor had almost no responses to hydrogen. As the temperature increased, the responses of the ZnO NPs gas sensor and the ZnO@ZIF-8 gas sensors to hydrogen gradually increased, and all of them reached the maximum at 290 °C. However, the sensors with growth time of 4 h and 8 h did not show superior responses to the ZnO NP gas sensor.

Figure 14 also shows the response coefficients of the ZnO NP gas sensor and the ZnO@ZIF-8 gas sensors to hydrogen at different temperatures. Compared with the responses, the influence of the growth time of the ZIF-8 film on the gas-sensing performance of the gas sensors can be clearly observed through the response coefficients. It is worth noting that the response coefficients to all the testing gases of the gas sensors with growth time of 4 h and 8 h were less than 1. When the growth time exceeded 8 h, the ZIF-8 films gradually thickened, and the response coefficients to hydrogen of the gas sensors were improved while the response coefficients to ethanol and acetone of the gas sensors were sharply dropped. Except for the sensors with growth time of 4 h and 8 h, the other sensors also showed their best working conditions at 230 °C–250 °C, and their response coefficients all showed a downward trend after 250 °C.

### 3.4. Discussion

The experimental results presented above showed that with the growth of ZIF-8, the selectivity of the gas sensors to hydrogen significantly improved. Simultaneously, it was observed that the responses of the gas sensors with growth time of 4 h and 8 h were reduced during the test. The mechanisms underlying these experimental results are discussed as follows.

**Figure 15:**
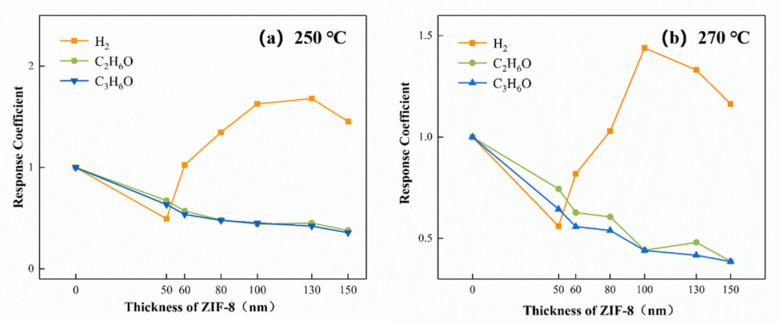
The response coefficients vs. the thicknesses of the ZIF-8 curve at (**a**) 250 °C and (**b**) 270 °C.

The molecular sieve effect of ZIF-8 is closely associated with the thickness of the membrane. The response coefficients of the sensors are critically affected by the thickness of the ZIF-8. Taking 250 °C and 270 °C as examples, the response coefficients vs. thickness curve is shown in Figure 15. With the extension of the ZIF-8 growth time, the thickness of the ZIF-8 film gradually increases, and the surface of the ZnO particles is tightly wrapped. The ZIF-8 film inhibits the responses of the sensors to ethanol and acetone. The thicker the ZIF-8 film is, the more obvious this inhibition is. For hydrogen, there is a peak in the response coefficients when the thicknesses of the ZIF-8 films varied. At 250 °C, when the thickness of ZIF-8 reaches 130 nm, the response coefficient of the 20-ZnO@ZIF-8 gas sensor to hydrogen is the best. However, when the thickness of the ZIF-8 film continues to increase, the response coefficients of the sensors to hydrogen decreases. At 270 °C, the peak of the response coefficients to hydrogen appear at the 100 nm ZIF-8 film. The results suggested that when the ZIF-8 film was too thick, the response coefficients could no longer be improved when the ZIF-8 film was grown to a critical thickness.

**Figure 16:**
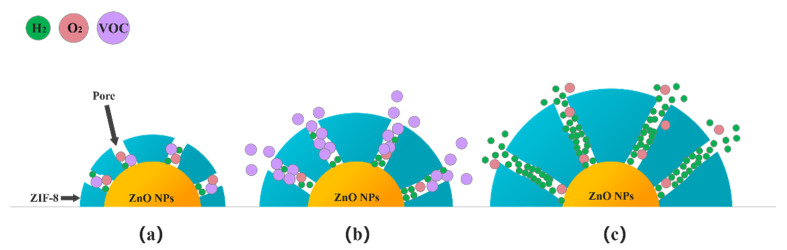
Schematic diagram of gas passing through the ZIF-8 films of different thicknesses: (**a**) Thinner; (**b**) Appropriate; and (**c**) Thicker.

The kinetic diameters of H_2_, ethanol and acetone are 2.89Å, 4.53 Å and 4.60 Å, respectively [39]. The standard pore diameter of ZIF-8 is 3.4 Å [33]. Theoretically, when a dense ZIF-8 film is formed on the surface of ZnO, only hydrogen gas with a molecular dynamics diameter of 2.89 Å will be allowed to enter the sensor. However, the pore size of the ZIF-8@ZnO prepared in this experiment is 11–12 Å. This may be due to the flexibility of the ZIF-8 structure itself and the influence of the Zn^2+^ content and distribution during the growth on the ZnO surface. However, in the microporous structure prepared in the ZnO@ZIF-8 composite, hydrogen still has the advantage of preferential contact with the ZnO NPs. The schematic diagram showing the influence of the thickness of the ZIF-8 film on the selectivity improvement of the gas sensors was illustrated in Figure 16.

**Figure 17:**
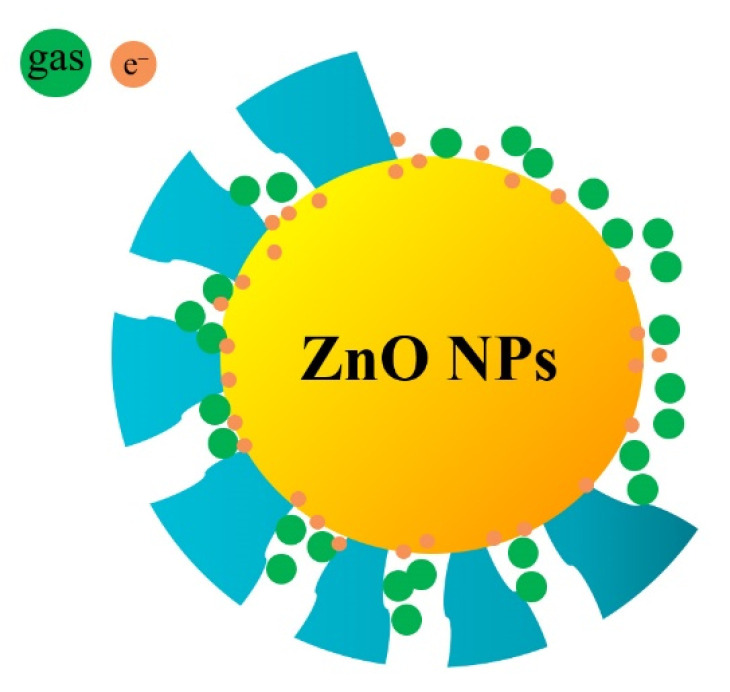
Schematic diagram of the ZnO@ZIF-8 composite with incomplete coating.

When gas molecules pass through the micropores in ZIF-8 zeolite, these micropores are not simply mechanically sieving for the gas molecules depending on their molecular dynamic diameters. This is not only a physical sieving process, as there are also interactions between gas molecules and the ZIF-8 zeolite. Although the size of the pores certainly affects the ease of passage of gas molecules, it cannot completely prevent the passage of gas molecules. For a gas molecule with a larger molecular dynamic diameter, the more obstacles it encounters when passing through the porous structure, the more difficult it is to quickly enter the micropores and make contact with the sensing materials. When ZIF-8 is thinner, as shown in Figure 16a, all gases can pass through the pores. In Figure 16b, when an appropriate thickness of the ZIF-8 film is formed, it is difficult for gases with larger kinetic diameters such as ethanol and acetone to pass through the pores smoothly, and they are adsorbed on the walls of the micropores. The molecular dynamics diameter of hydrogen is only 2.89 Å, which can prioritize other gases, even O_2_ in the air, to reach the surface of the ZnO NPs first. If the ZIF-8 film is too thick, the length of the micropores increases, which means that the gas molecules need to diffuse farther to react with the sensitive layer (Figure 16c). When hydrogen molecules pass through these long micropores, they will inevitably be adsorbed by the atoms on the micropore walls, resulting in the narrowing of the micropore’s diameter. It is difficult for subsequent hydrogen molecules to pass through these micropores and reach the surface of the sensitive layer. So the response to 1000 ppm hydrogen of the 24-ZnO@ZIF-8 gas sensor with a 150 nm ZIF-8 film is less than that of the 20-ZnO@ZIF-8 gas sensor with a 130 nm ZIF-8 film at 250 °C. The gas sensors with growth time of 4 h and 8 h do not form a complete core-shell structure when the growth time is short, which can be seen from the TEM results (Figure 7a,b). The gas molecules can freely contact and react with the uncoated ZnO NPs. This is why the 4-ZnO@ZIF-8 sensor and the 8-ZnO@ZIF-8 sensor behaved the same as the ZnO NPs sensor to hydrogen. The schematic diagram of the incomplete ZnO@ZIF-8 composite is shown in Figure 17.

## 4. Conclusions

In this paper, a kind of MOF (ZIF-8) was grown on the surface of the ZnO semiconductor material to improve the hydrogen selectivity of the sensors. First, nano-sized zinc oxide particles were prepared by a homogeneous precipitation method, and the sensors were prepared by screen printing technology. Then, a layer of ZIF-8 film was grown on the surface of ZnO NPs by hydrothermal synthesis to achieve the purpose of modification. The growth time of the ZIF-8 film was changed, and hydrogen (1000 ppm), acetone (50 ppm) and ethanol (100 ppm) were used as target gases. All the sensors were tested at 150 °C–290 °C. The optimal ZIF-8 film processing time for the hydrogen gas sensors was obtained at the optimal working temperature. When the thickness of the ZIF-8 film reached 130 nm, the sensor showed the best selectivity to hydrogen. Meanwhile, the sensor was restricted to detect the presence of gas molecules with large dynamic diameters. The selectivity improvement of the sensors with the ZIF-8 film is derived from the sieving effect of the micropores in the ZIF-8 and chemical adsorption of the atoms on the micropore walls. When the ZIF-8 film is too thick, it is hard for even the hydrogen molecules to pass through the micropores and they will inevitably be adsorbed by the atoms on the micropore walls, resulting in a decrease in the hydrogen selectivity of the sensors.

## Figures and Tables

**Table 1:** Symbols of the gas sensors.

Type	Processing Time/h	Symbol
ZnO NPs Gas Sensors	0	ZnO NPs
ZnO@ZIF-8 Gas Sensors	4	4-ZnO@ZIF-8
	8	8-ZnO@ZIF-8
	12	12-ZnO@ZIF-8
	16	16-ZnO@ZIF-8
	20	20-ZnO@ZIF-8
	24	24-ZnO@ZIF-8

## Data Availability

The date presented in this study are available on request from the corresponding author. The date is not publicly available due to privacy.
